# Sustainable urban system structure evaluation in sparsely populated areas: case study of the Qinghai-Tibet Plateau in China

**DOI:** 10.1038/s41598-022-20367-5

**Published:** 2022-09-27

**Authors:** Xuanxuan Shao, Yongling Yao

**Affiliations:** School of Applied Economics, Renmin University of China, Beijing, 100872 China

**Keywords:** Environmental social sciences, Socioeconomic scenarios, Sustainability

## Abstract

Urbanization in sparsely populated areas is critical for sustainability. The Qinghai-Tibet Plateau is a typical example of an ecologically fragile region that plays a crucial role in China’s ecological safety and water resource protection. We use a social network analysis to illustrate the structure of the urban system on the plateau and find that the agglomeration and diffusion capabilities of the core nodes are weak, which presents an obstacle to the sustainable development of the urban system. Nevertheless, we find that the intermediate nodes—which serve multiple integration functions for the various cultures, ethnic groups, and religions in the region—are core nodes that divide the dispersed cities and towns into four subgroups that function as small worlds (The cities and towns within one subgroup connect closely and each subgroup is organized independently somehow from others). Based on this finding, we suggest implementing a “double-layer” urban system to promote the sustainable urban development of the Qinghai-Tibet Plateau. This “double-layer” system breaks the usual urbanization trend, in which cities grow in size and their distribution becomes more concentrated; instead it provides a feasible way to maintain urban sustainability in a sparsely populated area.

## Introduction

Urbanization is the main driving force in regional development in developing countries (China e.g.). Since urban space is intensively agglomerated by human activities, cities and towns have a strong impact on the ecological environment, and urbanization can also cause great environmental pressure. A large body of research shows that urbanization with high population density has an important impact on the ecological environment, including climate, carbon emissions, and water and land resources^1,2^. Scholars predict that urban growth in this century will generate increasingly concentrated cities, some of which will grow enormously^3^. The sixth and seventh census data show that, urbanization growth in China during the past decade is 1.4% annually. Particularly, migration move mainly to big-sized cities and urban agglomeration areas. However, developing large-scale cities in sparsely populated areas, such as the Qinghai-Tibet Plateau, is not appropriate to foster big cities with densely populated distribution because of their fragile ecological environment.

The Qinghai-Tibet Plateau includes the total area of Tibet, parts of Qinghai, Xinjiang, Gansu, Sichuan, and Yunnan provinces in China. It covers an area of 2.5 million square kilometres, the largest plateau in China, with an average altitude of more than 4000 m, the highest in the world. The high altitude, low temperature, strong solar radiation, numerous rivers and lakes, widely distributed glaciers and permafrost, and rich biodiversity contribute to the fragility of the Qinghai-Tibet Plateau’s ecological environment^4^. In view of its unique geographical location and natural environment, the Qinghai-Tibet Plateau plays a critical role in the ecological safety and water resource protection of China, and even the world. Thus, protecting the ecological environment of the region must be a national and global strategy. The Qinghai-Tibet Plateau has long lagged behind in economic and social development; the plateau includes the typical impoverished areas in China. The aim of any development strategy for lagging areas in Western China will certainly be to promote the development of this area. Additionally, the region has a long history with cultural activities leaders, such as Fuxi, Emperor Yan, Lieshan, Gonggong, Siyue, Jintian, and Xia Yu, and all these cultures have now evolved into the plateau’s cultural system, which is dominated by Tibetans. The Tibetan cultural system is a typical part of Chinese culture, which will be impact by the modern cities. Therefore, ensuring sustainability and environmental protection is a significant challenge for the Qinghai-Tibet Plateau while respecting the ethnic culture and promoting economic growth.

The special geographical environment and historical background of the Qinghai-Tibet Plateau make its environmental system not only unique, but also closely related to global climate change. In the past decades, the rapid progress of urbanization in China has faced considerable challenges with respect to urbanizing the Qinghai-Tibet Plateau. Urbanization in the Qinghai-Tibet Plateau is not just a technical project of increasing the density and number of buildings and people, but it is also representative of the state and serves global sustainability. As a result, the evolution of urbanization in the Qinghai-Tibet Plateau will demonstrate a sustainable urbanization approach for other such special area. The urbanization process refers to increases in both the number of cities and their size, with the latter being more prominent than the former. In light of the significant differences among the natural environments within the Qinghai-Tibet Plateau, the residential spatial pattern has a disperse distribution; this pattern results in weak connections among the cities on the plateau and between the plateau cities and cities outside the region. This pattern characterized by sparsely populated area is not consistent with the trend of modern urbanization. Consequently, the overall function of this area is far greater than that of any of the individual cities within it. Hence, we will study the urban system, including all the main cities and towns associated with the overall urban function of the plateau. The objective of this study is to find a sustainable urban system for the Qinghai-Tibet Plateau to explore urbanization model at sparsely populated areas.

In terms of sustainable urban system, since a sustainable city is defined as a city where achievements in social, economic, and physical development are made to last and where there is a lasting supply of the natural resources on which its development depends^5^; The sustainable urban system refers that the natural ecosystem can carry the large-scale cities and the highly concentrated economic and social activities brought by the cities agglomeration. It is obvious that big-sized cities and urban agglomeration areas are not allowed at the Qinghai-Tibet Plateau due to the vulnerability of environment. However, the connected cities with multiple layers of nodes enable economies to concentrate knowledge, achieve greater efficiency in resource consumption (energy), higher productivity (GDP) and lower entropy (less CO_2_ emissions, better functional structure of the landscape)^6^; Particularly, the connected cities (towns) are more efficient for sustainable development than the isolated ones at sparsely populated areas. Additionally, urban sustainability is a multidimensional concept that includes environmental, economic, and social dimensions^7–9^. Then, the cities connectivity by economic, social, cultural and transportation spheres get greater efficiency instead of increasing few big cities at sparsely populated areas. On the other hand, by neglecting local heterogeneity, urban agglomeration policy risks exacerbating spatial inequalities in climate adaptation^10^. Thus, a gradual urbanization process must be adopted—one that relies on connectivity node cities—to conform to the geographical environment of the plateau^11^. As such, we employ a network analysis to explore the cities connectivity within the urban system. Additionally, to understand the full implications of the urban system, we investigate the different roles of cities in Tibetan cultural history^12^. We then infer that a rational urban system structure with special city nodes can improve the overall function of cities and towns network through spatial organization in the Qinghai-Tibet Plateau. This study will focus on these special nodes of the urban system network to find the sustainable urbanization process by their unique local relations.

To discover the evolution mechanism of the area’s urban system structure and identify the best strategy for future development, it is necessary to determine the influence factors of the urban system. Urbanization forces in the Qinghai-Tibet Plateau come from multiple sources, such as state aid investment, entry of enterprises to assist Tibet, and administrative expansion^13,14^. Hence, the urban system in the Qinghai-Tibet Plateau reflects the regions’ specific combination of natural environment, economic development, and social transition from a traditional society to a socialist one. The agglomeration and diffusion force of the core nodes are weak, which impedes the sustainable urban development. The intermediate nodes—which integrate multiple functions for the various cultures, ethnic groups, and religions in the region—are core nodes that arrange the dispersed cities and towns into four functional subgroups as “small worlds” with tightened their inner relation between cities and towns, also called cohesive subgroups in Social Network Analysis. Based on this finding, we suggest implementing a “double-layer” urban system to promote the sustainable urban development of the Qinghai-Tibet Plateau. The impacting factors on the urban network system will demonstrate the evolution mechanism of sustainable development in a sparsely populated area, and propose comprehensive sustainable development measures matching the Qinghai-Tibet Plateau, instead of focusing on simple urban or environmental sustainability, it also provides a representative case for other sparsely populated areas in the world.

The Qinghai-Tibet Plateau is a very typical physical geographic unit, and many studies have focused on the climate change, geological geography, ecological conservation, and environmental protection of the area^15,16^. Only a few studies on the Qinghai-Tibet Plateau refer to urbanization or social and economic development. The few urbanization studies that exist focus mainly on the region’s residential distribution and the forces responsible for the growth of cities. One study on residential distribution showed that the spatial distribution of the residential areas in the Qinghai-Tibet Plateau mainly depends on the natural environment^17^; therefore, there is a great difference in the density distribution of cities and towns around the plateau due to the various natural conditions. The specific spatial distribution of residents indicates few cities and towns, small urban scale, low urban development, large regional gaps, and weak economic relations among cities and towns^18^. However, the Qinghai-Tibet Plateau is not only characterized by its natural environment; it is also an area with extensive multi-ethnic integration. Moreover, as a specific strategic area, it receives a substantial amount of economic assistance from the central and local governments. Therefore, the urbanization path of the Qinghai-Tibet Plateau is quite different from that of other areas in China.

**Figure 1:**
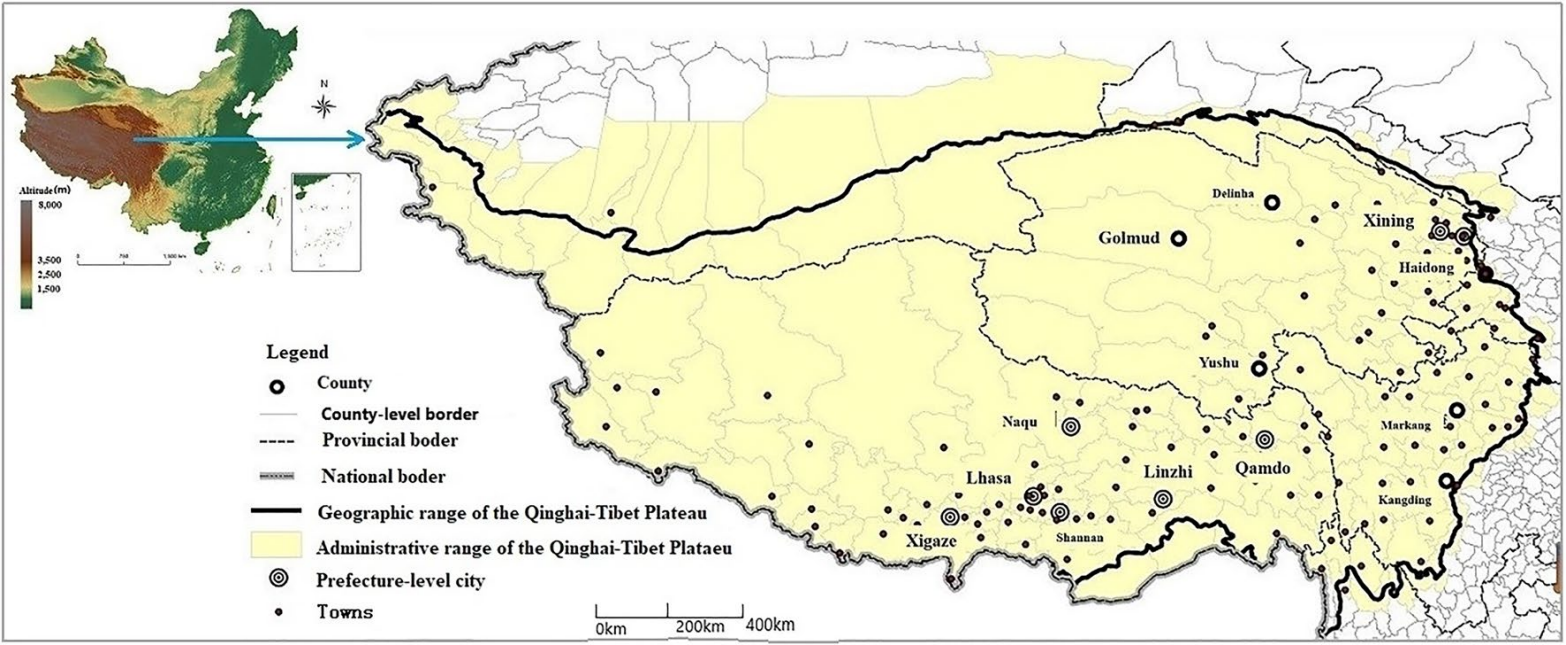
Distribution of selected cities and towns in the Qinghai-Tibet Plateau. Note: The map is derived from the National Science and Technology Infrastructure: ‘National Earth System Science Data Center’ (http://www.geodata.cn). All the spatial data are also the same. The southern boundary line indicates the schematic space range, not the actual national boundary. Besides Tibet and Qinghai, the areas included in the Qinghai-Tibet Plateau are Diqing Autonomous Prefecture bellowing to Yunnan province; Muli Autonomous County of Aba Autonomous Prefecture, Ganzi Autonomous Prefecture and Liangshan Autonomous Prefecture bellowing to Sichuan province; some autonomous counties of Wuwei, Zhangye, and Jiuquan cities in Gansu province; Gannan Autonomous Prefecture; and some towns in Hetian city and Hetian county in the Xinjiang Uygur Autonomous Region.

The Qinghai-Tibet Plateau has various definitions in different contexts. Sustainability requires maintaining the integrity of the ecological system^19^, from the southern edge of the Himalayas in the south to the northern edge of the Kunlun, Altun, and Qilian mountains in the north. The region comprises the Pamir Plateau and Karakoram mountains in the west. It is bounded by the southern or eastern feet of the Yulong Snow, Daxue, Jiajin, Qionglai, and Minshan mountains in the east and is connected with the western section of the Qinling mountains and Loess Plateau in the east and north-east^20^. Considering the relative integrity of the administrative units, we define the Qinghai-Tibet Plateau as including the entire Tibet Autonomous Region and Qinghai province, as well as some autonomous prefectures and counties in Yunan, Sichuan, Gansu provinces, and Xinjiang Uygur Autonomous Region respectively. Thus, the region of Qinghai-Tibet Plateau is composed of a total of 17 prefectures and 169 administrative cities and counties, covering 375 nodes (see Fig. 1 for their distribution).

**Figure 2:**
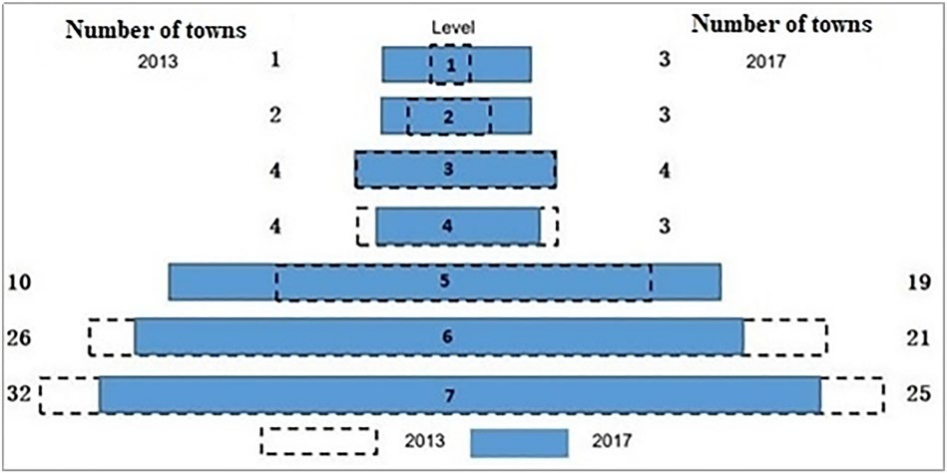
Pyramid structure of the urban system in the Qinghai-Tibet Plateau (2013 vs 2017).

The mainstream of urbanization is population urbanization. The urban system is mainly illustrated by the population size cities. Then the urban system structure is also constructed by the population sizes of cities and towns. The level number of pyramid to illustrate urban system structure is flexible according to the population sizes of cities and towns. The typical pyramid structure normally has ten levels (A–J) which includes world city, national capital, regional centres, etc. from top to bottom. As the top city at the Qinghai-Tibet Plateau, Xining is the capital city of Qinghai province which corresponds to the D level of the typical urban system and leads to the missing of A–C levels. Then, we divide the selected urban units from D to J into seven levels for the urban system at the Qinghai-Tibet Plateau. To avoid cities and towns of similar sizes being classified into different levels by artificially set critical values, we adopt the natural fracture method to obtain the critical population values for each level. The division criteria are as follows: ≥ 300,000, 103,387–300,000, 52,635–103,387, 24,815–52,635, 10,391–24,815, 4,250–10,391, 1,195–4,250 (corresponding to more than 300,000, 100,000–300,000, 50,000–100,000, 25,000–50,000, 10,000–25,000, 5,000–10,000, and 1000–5000; see Fig. 2).

The Fig. 2 shows that both The fastest-growing number of cities (towns) is in the fifth level, followed by the first and second levels, and the number of cities in the fourth, sixth and seventh levels is decreasing. This indicates the middle-level cities (the fifth level e.g.) very unique and they played important role in the urban system at the Qinghai-Tibet Plateau. However, previous studies have mapped the urbanization process in the area^21,22^. They have explored the reasons for the region’s low level of urbanization by comparing its urbanization process with the level and structure of typical urbanization processes, which focus on the role of core cities^23–25^. These studies concluded that lagged economc level and sparsely population distribution made the low level of urbanization. However, these conclusions are too general without conforming to the specific geographical environment and social-cultural situation of the Qinghai-Tibet Plateau.

The aim of this study is to investigate the role of middle-level towns in the urban system evolution of sparsely populated areas and find a way to promote an urban system structure while maintaining the sustainability of the Qinghai-Tibet Plateau by emphasizing the development of middle-level towns. We illustrate the network structure using the social network analysis method and use it to determine the role of middle-level towns in the network. We then use this to propose an urban system for the Qinghai-Tibet Plateau based on the role of middle-level towns. The goal of this paper is to give a new way to understand the role of middle-level nodes in the urban system connectivity for sparsely populated area that is quite different from the typical ones in developed areas.

## Results

### Network structure of the urban system in the Qinghai-Tibet Plateau

We set up the connections between cities and towns with gravity model and show the multiple dimensional perspectives of the urban system structure using Social Network Analysis method by calculating various parameter values with the software—Ucinet (see the details in the “Method” section). We also use this to ascertain the status of nodes in the structure to identify the role of middle-level towns. Further, we consider the internal subgroup characteristics to demonstrate the subgroup organization of the middle-level towns.

#### Parameters of the overall network structure

The parameter values of the overall network structure indicate the whole function of all the nodes in the urban system structure. We use the method of calculating parameter values of overall network structure, which includes the values of network density, the centrality, out-centrality, in-centrality and intermediary centrality are calculated by formulas (3)–(6), as shown in Table 1.

**Table 1:** Values of the overall network structure.

Index	Value	Range
Network density	0.151	[0,1]
Centrality	0.245	[0,1]
Out-centrality	0.242	[0,1]
In-centrality	0.281	[0,1]
Intermediary centrality	**0.448**	[0,1]

Significant values are in [bold].

**Figure 3:**
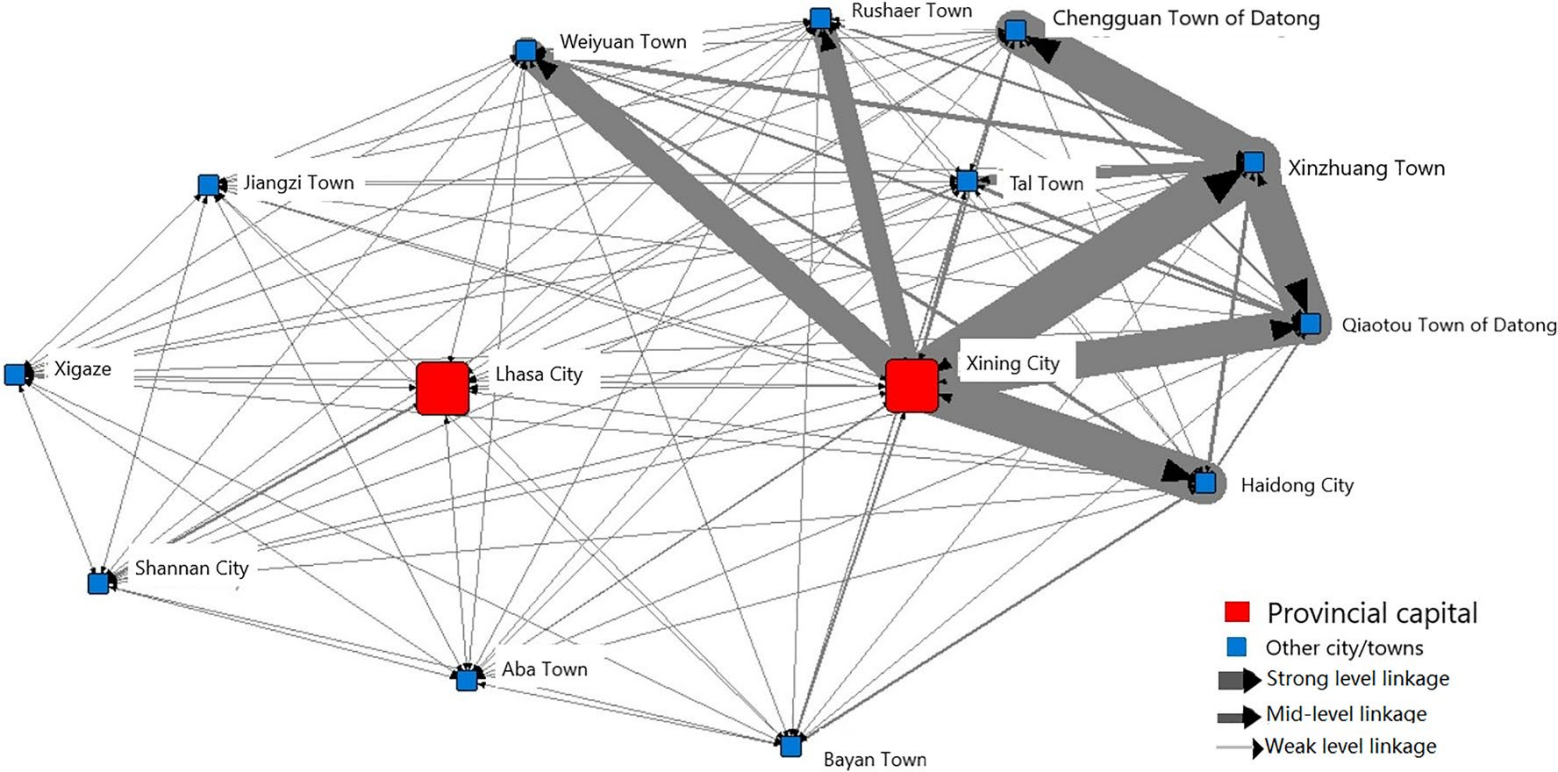
Main network linkage of the cities and towns in the Qinghai-Tibet Plateau.

Table 1 shows that the network density is only 0.151, much lower than a normal network density value(We calculated the density values of networks by population in the Jinjinji area, the Yangtze River Delta area, the Pearl River Delta area, north-eastern China, the Yellow River Basin area, and the Middle Reaches of the Yangtze River area, among others. The density values of their network areas mostly ranged from 0.2 to 0.5. The biggest value was 0.608). This indicates that the connection among cities and towns in the Qinghai-Tibet Plateau is extremely weak. The centrality is only 0.245, which is also low, and the centre-oriented trend of the urban network is not obvious. However, the in-centrality value is slightly larger than the out-centrality value, indicating that the agglomeration force is greater than the diffusion force, and populations are slowly moving from the outer cities or towns into the core ones. The most notable result is that the intermediary centrality value is much higher than the other parameters. This indicates that the role of intermediate nodes is much more significant to the whole function of the urban system structure network. In a hierarchical urban scale system, large-sized cities are in the core position, and smaller ones are in marginal positions. As such, the cities or towns at the medial level would play a decisive role in connecting the lower- and higher-level cities or towns in the Qinghai-Tibet Plateau. We use natural break method again, to divide the linkages into 7 levels, and select the strongest linkage lines to draw a linkage picture for the cities and towns in the Qinghai-Tibet Plateau (see Fig. 3).

Figure 3 shows that the densest linkages are among the cities and towns in the north-eastern corner of the plateau, where the most developed areas and the biggest cities are located, namely Xining and Haidong. The next highest density of link lines is distributed around Lhasa, which connects with Xigatse and Shannan. These are all prefectural cities. The rest of the nodes on the plateau seldom associate with each other (their link values are lower than average). This figure further demonstrates that simply organising the urban system by the cities on the Qinghai-Tibet Plateau is not sufficient.

#### Parameters of the individual nodes in the network

The parameter values of the individual nodes reflect the role of each city or town in the network. We use the method of calculating *parameter values of individual nodes.* The real parameter values and their standardised values from the formulas (7)–(10) are shown in Table 2. To reveal the status of urban nodes in the network more clearly, being too much to list all the nodes, the top ten cities and towns for each of the three centrality values are listed in Table 2.

**Table 2:** Parameter values of the individual nodes in the Qinghai-Tibet Plateau.

City or town	In-node-centrality	City or town	Out-node-centrality	City or town	Intermediary node-centrality
Xining (Qinghai)	33 (0.43)	Baohe town (Yunnan)	30 (0.39)	**Labrang town (Gansu)**	**1426.99 (0.24)**
Haidong city (Qinghai)	31 (0.40)	Aba town (Sichuan)	29 (0.38)	**Aba town (Sichuan)**	**1179.77 (0.20)**
Qiaotou town (Qinghai)	31 (0.40)	Mangqu town (Qinghai)	25 (0.32)	**Centre town of Zhouqu (Gansu)**	**1104.30 (0.19)**
Jishi town (Qinghai)	30 (0.39)	Ping'an town (Qinghai)	24 (0.31)	**Heyin town (Qinghai)**	**908.37 (0.16)**
Xinzhuang town (Qinghai)	30 (0.39)	Bayan town (Qinghai)	23 (0.30)	Centre town of Lintan (Gansu)	554.23 (0.09)
Weiyuan town (Qinghai)	29 (0.38)	Heyin town (Qinghai)	23 (0.30)	Liulin town (Gansu)	547.83 (0.09)
Chuankou town (Qinghai)	27 (0.35)	Tianjiazhai town (Qinghai)	23 (0.30)	Chuankou town (Qinghai)	361.06 (0.06)
Bayan town (Qinghai)	26 (0.34)	Hongwansi town (Gansu)	23 (0.30)	Bayan town (Qinghai)	351.18 (0.06)
Guanlongkou town (Qinghai)	26 (0.34)	Chahanwusu town (Qinghai)	23 (0.30)	Guanting town (Qinghai)	343.80 (0.06)
Lushaer town (Qinghai)	25 (0.32)	Xiaoxia town (Qinghai)	22 (0.29)	Gaocheng town (Sichuan)	341.76 (0.06)
Maximum value	33 (0.43)	Maximum value	30 (0.39)	Maximum value	1426.99 (0.24)
Minimum value	0 (0)	Minimum value	1 (0.01)	Minimum value	0 (0)
Mean	11.61 (0.15)	Mean	11.61 (0.15)	Mean	149.67 (0.02)

Note: Standardized values are in parentheses. The names in parentheses are the provinces in which the city or town is located.

Significant values are in [bold].

Table 2 shows that the mean in-degree and out-degree values are very small, indicating that in the 78 research units in the Qinghai-Xizang Plateau, the population agglomeration and diffusion forces are weak. However, the values of intermediary centralities are prominently high. This further underscores the significant role played by the intermediate nodes in the urban network of the Qinghai-Xizang Plateau. Nevertheless, the intermediary centrality values vary significantly, and only a few of the intermediate nodes show a high value for intermediary centrality. This indicates that only some special towns (top four) can play a critical role in the urban network because of the fragile natural environment and the unbalanced socio-economic development.

Table 2 shows that the top ten in-node-centrality cities or towns are all located in Qinghai province. This indicates that the cities or towns in Qinghai province have an obvious agglomeration force in the network. The top ten out-node-centrality towns are distributed around Yunnan, Sichuan, Gansu, and Qinghai provinces and are mainly spread across the borders of these provinces. This shows that the populations in these marginal areas tend to migrate to the core cities and towns. The top ten towns in terms of intermediary centrality are located around the border between Qinghai, Gansu, and Tibet, where it is difficult to connect with the core cities and towns. The function of intermediary nodes in the urban network, however, helps establish some connections, which further reveals the importance of these intermediate nodes in the urban network of sparsely populated areas.

Among the top ten intermediate nodes, the intermediate roles of Labrang, a town in Gansu province; Aba, a town in Sichuan province; the central town of Zhouqu in Gansu province; and Heyin, a town in Qinghai province are all very prominent. Labrang, a town in Xiahe county, Gansu province, is famous for the Labrang temple. It has been the Buddhist activity centre of Gansu, Qinghai, and Sichuan provinces since ancient times, and it is one of the six patriarchal temples of the Gelu sect of Tibetan Buddhism as well as the Sino-Tibetan trade centre. The role of Labrang in the urbanization of the Qinghai-Tibet Plateau is completely consistent with the conclusion of a study on ‘monastery’ urbanization^26^. Aba, as the central town of the Tibetan and Qiang ethnic autonomous prefecture in Sichuan province, connecting some parts of Sichuan province with the Tibet area, undertakes the task of multi-ethnic integration. Zhouqu, located in the southern part of Gansu province, which is from a Tibetan autonomous region. It has a long history of connecting people in Gansu province with those in the Tibet area, and it reflects the characteristics of transforming from a Qinghai-Tibet Plateau area to an inland area. Heyin is on the border of Xining (the provincial capital of Qinghai) and is home to various ethnic groups, including Han (72.98%), Tibetan (11.95%), Hui (1.62%), and Tu, Sala Mongolian (3.45%). Heyin plays the role of an intermediate centre of multi-ethnic interactions, connecting Xining with other distant centres. The scenarios of the intermediate towns discussed above show that location and ethnic activities are the main reasons for their intermediate roles in the urban system of the Qinghai-Tibet Plateau. Thus, ethnic activities are essential for connecting people in sparsely populated areas.

### Internal subgroup structure of the urban system

Due to the scattered distribution of cities and towns in the Qinghai-Tibet Plateau, there must be valid internal subgroups that serve as small, independent worlds within the larger network. We use the method of CONCOR to define the nodes into the subgroups. These subgroups represent small worlds; that is, the residents typically live and interact within these clusters, without venturing out. The specific steps for identifying these subgroups involve interactively calculating the correlation coefficient of each node and then grouping nodes with similar correlation coefficients together. To further observe the core-periphery structure of the urban system, we divide the nodes in each subgroup into two types: core nodes (centrality values are higher than the mean value of the subgroup) and periphery nodes (centrality values are lower than the mean value of the subgroup; see the results in Table 3).

**Table 3:** Four subgroups in the Qinghai-Tibet Plateau.

No	Core nodes	Periphery nodes	Network density	Notes
Subgroup 1	Haidong city Labrang town Huazangsi town	13 towns	0.463 (25)	Total 22 towns; 15 towns included in Haidong city
Subgroup 2	Xining city Heyin town	16 towns	0.488 (29)	Total 26 towns; 18 towns included in Xining city
Subgroup 3	Xigaze city Shannan city Lhasa city	5 towns	0.333 (6)	Total 9 towns
Subgroup 4	Aba town Ganzi town	7 towns	0.433 (19)	Total 21 towns distributed around two prefectures

Note: The values in parentheses are the mean values of centrality of each subgroup.

Table 3 shows that the core nodes of each subgroup are the cities and towns with high values of intermediary node-centrality. In subgroup 1, the core nodes are Haidong city, Labrang town, and Huazangsi town. Among these, Haidong city is one of the two prefecture cities in Qinghai, and it is also the node connecting Qinghai and Gansu provinces. It belongs to the transitional mosaic zone from the Loess Plateau to the Qinghai-Tibet Plateau. Labrang town has the highest intermediary node-centrality value in the Qinghai-Tibet Plateau, as the fifth level cities (towns) displayed in the pyramid structure of above. Huazangsi town is famous for the Huazang Temple, which has more than 600 years of history. In subgroup 2, the core nodes are Xining city and Heyin town. The former is the capital of Qinghai province and the central city of the Qinghai-Tibet Plateau; the latter has the fourth highest intermediary node-centrality value in the Qinghai-Tibet Plateau, as indicated as above. In subgroup 3, the core nodes are the three prefecture cities in Tibet, which are the centres of the southern and western parts of Tibet, respectively. In subgroup 4, the nodes are all towns belonging to Aba and Ganzi prefectures, with the central towns of Aba and Ganzi being the core nodes. Of these, Aba has the second highest intermediary node-centrality value in the Qinghai-Tibet Plateau. All the above, other than the central cities, demonstrate the significant importance of intermediary node-centrality to the small worlds of urban systems.

The distribution of the four subgroups demonstrates the small worlds of the urban system of the Qinghai-Tibet Plateau. Subgroup 1 is located primarily at the border of Gansu and Qinghai provinces—an area with lower altitude and better natural conditions than those of the other three subgroups. It is an intersection area of farming and pastoral cultures, with Hui, Tibetan, Tu, Salar, and Mongolian ethnic populations. These groups created the ‘Hehuang culture’, a unique cultural mix with agricultural, commercial, handicraft, and cultural exchanges. It is an important branch of the famous ‘ethnic corridor’^27^, Subgroup 2 is located on the eastern edge of the Qinghai-Tibet Plateau, covering a relatively wide range of territories and a large number of ethnic minorities, mainly Hui, Tibetan, and Tu populations, where Buddhism, Islam, Taoism, Christianity, and Catholicism coexist. The ancient ‘Silk Road of the Qinghai-Tibet Plateau’, which has existed since the Tang dynasty and has connected the Qinghai-Tibet Plateau and the inner land of China, runs through this area^28^. Subgroup 3 is located in southern Tibet and the southwest of Xinjiang and has minimal natural resources. They both belong to the border of the urban system of the Qinghai-Tibet Plateau and are quite different from other places. Subgroup 4 covers two autonomous prefectures in western Sichuan province, namely Ganzi and Aba. This subgroup is characterised by intersecting between farming and animal husbandry^29^. Thus, the distributions of the four subgroups show that these small worlds of cities and towns are grouped by their various natural environments, cultures, and religions. Each subgroup requires its specific centre nodes to provide a connection among its members, which means they function as the sub-urban system. These four subgroups, organized together, constitute the whole function of the urban system in the Qinghai-Tibet Plateau.

### Influencing factors on the urban network structure in the Qinghai-Tibet Plateau

Owing to the dispersion and particularity of distributions, it is necessary to organise the urban system structure in sparsely populated areas specifically and carefully. This makes it critical to discover the factors influencing the urban network structure, based on which the urban system can be planned efficiently and rationally. As mentioned at the introduction section, sustainable urban system with the cities connectivity by economic, social, cultural and transportation spheres get greater efficiency instead of increasing some big cities at sparsely populated areas; Urbanization forces in the Qinghai-Tibet Plateau come from multiple sources, such as state aid investment, entry of enterprises to assist Tibet, and administrative expansion except for the geographical status of the plateau and transportation. We select the driving factor as shown in Table 4. The Altitude gradient indicates the height range between cities, Geographical proximity reflects the spatial distribution, both are the special geographic features at the Qinghai-Tibet Plateau; Transport accessibility is essential for all the connectivity; Administrative border denotes the Chinese institutional system; Difference in the number of enterprises indicates the entry of enterprises to assist Tibet by the state and local governments aid investment. The dependent variable, urban system connectivity, is constructed by urban population network which is calculated by gravity model. Then we use the Quadratic Assignment Procedure regression (see methods for the retails) to find the influencing factors. The correlation coefficient and significance of each independent variable to one another is obtained (see Table 4).

**Table 4:** Quadratic assignment procedure results on the influencing factors of urban population network structure.

Independent variables		Urban population network structure		
Influencing factors	Non-standardised coefficient	Standardised coefficient	P-value	Standard deviation
Altitude gradient	0.0116	0.1513***	0.0001	0.00158
Geographical proximity	0.2312	0.2321***	0.0001	0.02057
Transport accessibility	0.1323	0.1579***	0.0001	0.0186
Administrative border	0.2517	0.1559***	0.0001	0.03062
Difference in the number of enterprises	0.00002	0.0076***	0.0001	0.00007
R square	0.301			
P-value	0.000			
Samples	6006			
Random permutations	10,000			

Table 4 shows that the influence of geographical proximity and altitude gradient on the population network structure is significantly positive, and the standardised coefficient value of the former is higher. This indicates that the natural conditions of the Qinghai-Tibet Plateau are still the most important factors affecting the urban system. After geographic proximity, transport accessibility has the most significant positive impact, beyond the limitations of local administrative borders and altitude. This indicates that the population network of the Qinghai-Tibet Plateau is closely related to the transportation network. Improvement of transportation conditions is conducive to reshaping the scale structure of cities and towns in this area. In terms of local administration, the boundaries of the administrative area have a significant impact on the urban system, which is consistent with the situation we observed in the subgroups. Note worthily, although the number of enterprises representing the economic factor is significantly positively related to the network structure of population size, the influence is relatively limited and does not determine the spatial distribution of population on the Qinghai-Tibet Plateau. This indicates that the role of economic forces is not a key factor in the formation of the urban system in a sparsely populated area; this is completely different from the situation in the eastern and central regions in China, where there exists a mutual promotion of the economy and urbanisation.

## Discussions

As the typical sparsely populated area, the special natural environment, complex ethnic relations, diverse cultural and religious traditions, and other social features together determine the unique urban system of the Qinghai-Tibet Plateau. For the network structure at sparsely populated areas, with the rapid development of urbanization, the scale structure of the urban system on the plateau shows that the number of low-level towns has decreased, the number of high-level towns has increased significantly, and the urban systems have demonstrated a trend of gathering upward. It is difficult to maintain urban sustainability in sparsely populated areas. However, the significant role of middle-level towns in the evolution of the urban system structure makes the different sustainable urbanization pattern in the Qinghai-Tibet Plateau, which is different from the one at densely populated areas.

For comprehensive sustainable approach which concerning the complexity of nature, ethnic culture and urbanization, further findings regarding the individual nodes show that these middle-level towns can function as intermediaries and are very likely to be the intermediate nodes in the urban network. These intermediate nodes represent integration local centres for the cultural and religious activities of various ethnic people. These nodes are special links that satisfy the various requirements of different peoples, and they serve as bridges among the small towns distributed at the border and big cities located in core areas of socio-economic development. This specific sustainable urbanization based locally on the specific natural environment and social conditions at the Qinghai-Tibet Plateau is suitable for sustainable development. For other sparsely populated areas, natural, social and cultural linkages except for agglomeration would be the connections to organize their urban systems.

The term urban system refers to the structures and relationships among cities and towns. With the increasingly strengthened links among cities and towns, studies on urban systems have begun focusing more on the connections among node cities and towns. Only by optimizing the spatial connections among these cities and towns can we form a comprehensive and effective urban system—one that gives full play to the overall advantages of the various cities involved^30^. However, scale structure can only show the distribution of cities and towns of different sizes; the internal relationships among these units and the factors that affect these relationships cannot be identified. As a new spatial organization pattern, networks can make up for the defects of the central place model^31^ and capture the process of urban development, thus reflecting the structural effects of urban systems^32^. In the past two decades, urban network model has gradually become the mainstream paradigm of urban system research in China^33^. A network not only reveals the relationships among cities and towns, but also reflects the attributes of the geographical space of the connections. This is because these kinds of connections are related to their geographic attributes and spatial distances of these nodes. Additionally, cities connectivity is the main determinant of its role in the future development of the urban system, its structural characteristics can also indicate the urban system’s trend when using the urban scale index to build the network structure. Particularly, at the sparsely populated areas, the cities connectivity is more important than the cities’ size because isolated small city is less efficient and less sustainable than the connected cities. However, the actual city network of the Qinghai-Tibet Plateau is still being formed; the internal relationships are still critical for the urban system. This situation leads researchers to ignore network connections and focus on city size at this kind of areas. We use this network method to demonstrate the formation mechanism of the urban scale structure in the Qinghai-Tibet Plateau to display the unique structure which is different from the typical ones at densely populated areas.

Our study finds that these intermediate nodes are especially important for maintaining a connection among the cities and towns and ensuring the function of the urban system as a whole in terms of cultural, ethnic, and religious activities. As introduced at the introduction, the sustainable urban system is the composite system with multiple dimensions of natural, economic, social and cultural issues. For sparsely populated areas, the sustainable urban system denotes the connections between cities instead of cities size and agglomeration. The “double-layer” with the special intermediate nodes meets the sustainable urban system at the Qinghai-Tibet Plateau. Based on this finding, we suggest planning a sustainable “double-layer” urban system in this sparsely populated area. Specifically, the upper layer is composed of the top-level cities, which are the core of the whole urban system. These cities connect both with each other and with the sub-core nodes. The lower layer is the intermediate nodes, which consist of middle-level towns or towns that serve multiple functions, integrating these parts into the whole urban system of the Qinghai-Tibet Plateau. A “double-layer” urban system provides a new way to promote urban sustainability in sparsely populated areas by breaking the usual urbanization trend in which cities grow and the distribution becomes increasingly concentrated.

In terms of the mechanism analysis for the factors influencing the network structure, the influence of economic factor is non-significant, that is different from densely populated areas. We further explore which of the independent variables affect the role of the economy on urban development. Using the gravity model and network analysis we mentioned in the “Method” section, we construct an enterprise difference matrix among cities and towns and use the mean value of the row as the threshold value to construct the dependent network variable. We continue to consider the effects of altitude gradient, geographical proximity, transport accessibility, and administrative border on economic factors, and we use the same regression method to test the robustness of the results above (see Table 5).

**Table 5:** Quadratic assignment procedure results on the influencing factors of the urban enterprise network structure.

Independent variables		Urban enterprise network structure		
Influencing factors	Non-standardised coefficient	Standardised coefficient	P-value	Standard deviation
Altitude gradient	**− **0.0024	− 0.0554*	**0.0798**	0.0020
Geographical proximity	**− **0.005	**− 0.0088**	**0.4723**	0.0249
Transport accessibility	**− **0.0065	**− 0.0137**	**0.40906**	0.0227
Administrative border	0.0949	0.1031**	**0.0190**	0.0336
R square	0.009			
P-value	0.010			
Samples	6006			
Random permutations	10,000			

Note: ***, ** and, * indicate that the results are significant at the 1%, 5%, and 10% confidence levels, respectively.

Significant values are in [bold].

Table 5 shows that the positive correlation between the number of enterprises and the administrative boundary is the most significant, with the highest coefficient. There is also a significant negative correlation with altitude, which is weaker than the effect of administrative border. This shows that the effect of economic development on the urban system structure works to a certain extent through administrative forces and depends on the natural conditions. However, the coefficients of the number of enterprises with transport accessibility and geographical proximity are not significant, indicating that the choice of location for enterprises in the Qinghai-Tibet Plateau does not follow the universal rule wherein enterprises are generally located in areas close to one another and with good traffic conditions. As administrative power plays an important role in the area’s economic development; and the region’s urban development depends highly on national policy. These findings are consistent with the conclusions of previous studies and further clarify that urbanization in the Qinghai-Tibet Plateau is not just a technical project of increasing the density and number of buildings and people, but it is also representative of the state and serves global sustainability.

In terms of the methodology of network, there are couples of ways to display the relations between nodes. Social Network Analysis (SNA) is the most useful tool to illustrate the features of network structures by various parameter values from a multiple dimensional perspectives, such as centrality, network density and network cohesion index, etc. Because these parameter values are calculated by the connecting matrix among nodes, the network structure relies on the matrix data to reflect the connectivity between nodes. Defining the connectivity by data is too numerous to enumerate which lead the results unconformity. The data we used from transportation lines, economic issues, etc. are objective statistics that can avoid this defect. However, the connectivity features calculated by SNA are just 0 or 1 instead of degree of relations which covers the details of connectivity. The network method reflects three points for illustrating urban system structure. (1) The relations between cities are multiple dimensions and reinforce each other, which can be used to understand the urban system evolution essentially; (2) The attractive force is asymmetric between cities scale sensitively; (3) The urban system network is horizontal and vertical structure which can be explained the combination of the Central place theory and the Central flowing theory. The future of urban system network indicates that: (1) With new techniques of network tools are developed in the future and more spatial–temporal big data involved into setting up connecting matrix, more detailed urban system, for example, the polycentric form of urban system can be identified and measured to display the “double-layers” structure. (2) This study illustrates the urban system structure with social network analysis that just display the general structure pattern as the start of “double-layer” urban system. The further study about internal structure and the urbanization mechanism by more motivations reinforcing each other need complicated technics, such as super network, K-shell decomposition.

## Conclusions

This study uses social network analysis to illustrate the urban system structure of the Qinghai-Tibet Plateau. The analysis finds that the agglomeration and diffusion capabilities of the core nodes are weak, which has become an obstacle to the sustainable development of the urban system in this sparsely populated area. However, one interesting finding is that intermediate nodes, which function as areas of integration for the various cultures, ethnicities, and religions in the area, are the core nodes. These nodes divide the dispersed cities and towns into four small subgroups, which essentially function as small worlds. These subgroups are located in the north-eastern, eastern, southern, and western parts of the plateau, respectively. These four sub-urban systems consist of core cities and intermediate nodes.

The findings denote three benefits for the urban system evolution. Firstly, sustainable urban system is a complex system which needs the integration of economic, social, cultural and ecological activities together. The social and cultural factors are more important for sustainable urban system at sparsely populated areas than that of densely populated areas. The policy makers should pay more attention on the social and cultural issues instead of economic ones for sustainable urbanization at sparsely populated areas. Secondly, the connectivity between cities is the effective way to get more efficient than the isolated city, which is the coincident for all the urban systems. Particularly, the connectivity is more efficient for the sustainable urban system at sparsely populated areas than the ones at densely populated ones. Because the economic connectivity between cities at developed areas depends on market; however, the social and cultural connectivity between cities are promoted by the local governments and organization at sparsely populated areas. This implies that the different connective ways of sustainable urban system at sparsely populated areas from the ones at developed areas. Thirdly, sustainable urban system is local heterogeneity at sparsely populated areas case by case. The “double-layer” urban system is provided by observing the pyramid structure, the role of intermediate nodes and the four sub-groups of the network. There are different sustainable urban system structures for other kinds of areas.

The result suggests that policymakers should build sustainable urban system upon intermediary nodes rather than pursue traditional urban development goals, and further research is needed to identify best practices for implementing and enhancing “double-layer” systems in sparsely populated areas, such as desert areas and mountainous areas, where population distribute scattered and is impossible to agglomerate big cities because of separated location and bad natural environment.

In terms of the underlying mechanism, natural conditions are the most important factors determining the urban system in the Qinghai-Tibet Plateau. This indicates that the natural condition is still the critical determinant in this sparsely populated area. To maintain sustainable urbanization, it is preferable to select an effective urban structure based on local natural conditions instead of increasing the size of city and agglomerating the urban system in sparsely populated areas. In terms of crafting a “double-layer” urban system, the most direct way to improve the connections among nodes is to construct transportation facilities. This would be an efficient tool to improve the network function of the dispersed nodes—other than increasing direct economic investment. Further, the results of the influencing factors show that the administrative force is powerful in the development of intermediate nodes in sparsely populated areas. We hope that cooperative urbanization that surpasses administrative boundaries would be sufficient to maintain valid urban system structures in other sparsely populated areas globally. This results imply that, as the state strategy, urbanization at the sparsely populated areas require multiple dynamics instead of economic force; require spatial cooperation other than the motivation by region.

## Method

To highlight the role of cities and towns as population centres and eliminate the interference of small-sized towns, we select towns with a population of more than 1,000 residents and a density of more than 100 people per square kilometre in 2017 as the objective nodes. For Xining, Haidong, and other prefecture-level cities, we use their municipal districts as sample units to maintain the consistency of the spatial scope. The final number of population-centre samples is 78, including 45 in Qinghai, 18 in Sichuan, 6 in Gansu, 4 in Tibet, 4 in Xinjiang, and 1 in Yunnan (see the supplementary table S1 for the names list).

The data, including population and the numbers of each city or town included are derived from the ‘County Statistical Yearbook (Township Volume) 2014’, China Urban Statistical Yearbook 2014, ‘County Statistical Yearbook (Township Volume)2018’, and ‘China Urban Statistical Yearbook 2018’ repositories.

[https://chn.oversea.cnki.net/kcms/detail/detail.aspx?dbcode=CYFD&filename=N2015040001000006&dbname=CYFDLM].

Altitude gradient, Administrative bounder, Geographic proximity and Transport accessibility are from The minimum of traffic time between towns was captured by Gaode-Map software through python programming, which are available from the corresponding author on reasonable request.

The analytical tools are Ucinet 5.6 and ArcMap 10.2.

### Network method

#### Establish the relation matrix among cities and towns

The general principle for the relation is that the larger the urban size and the shorter the distance, the greater the influence on other cities and towns and the higher the status in the urban system. Referring to Newton’s universal gravity model in spatial analysis, gravity models with various situations have been used to represent the relations between spatial units. Due to the asymmetric functions between bigger cities and the smaller ones, we use the asymmetric gravity model to calculate the relations between cities (towns) one by one and construct the asymmetric matrix relations. The calculation formula is as follows:

1 $$P_{ij} = K_{ij} \frac{{Pop_{i} Pop_{j} }}{{d_{ij}^{2} }},$$

2 $$K_{ij} = \frac{{Pop_{i} }}{{Pop_{i} + Pop_{j} }},$$

where ${P}_{ij}$ is the comparative structure coefficient of the population size of city *i* and city *j*, ${d}_{ij}$ is the spatial distance between the two places, ${Pop}_{i}$ and ${Pop}_{j}$ represent the resident population in the established area of city *i* and city *j*, respectively, in the Qinghai-Tibet Plateau. ${K}_{\mathrm{ij}}$ represents the structure coefficient of city *i* to city *j*. Since the population sizes of city *i* and city *j* are different, this correction coefficient shows an asymmetrical structure among the cities and towns in the Qinghai-Tibet Plateau. The mean value of the P_ij_,(j = 1,2,…,n, j ≠ i) , P_i_ is the mean value of the P_ij_ and used as the threshold to judge whether there is a correlation structure between city *i* and city *j*. We obtain the asymmetric urban network based on population size.

#### Calculate parameter values of overall network structure

There are three kinds of network features: overall network, individual nodes, and internal subgroups. The feature of the overall network structure is reflected by the Network Density and the Centrality of all nodes. Based on the definitions of John Scott (2000) about the features of social network analysis, we write all the formulas to calculate the parameters^34^. Actually, all the calculaitons are embedded in the calculating progress of Ucinet software.

*The Network Density* indicates the structural compactness among the nodes within a network, which is reflected by the ratio of the actual number of associations to the maximum number of possible associations in the whole network. The higher the value, the more compact the structure is. Formula is not necessary.

*The Centrality indexes* represent the direct connection of one node with multiple other nodes in the network. With an asymmetric network, out-centrality indicates the direction of association from the central points to the outer ones, which represents the spreading function of the central nodes in the network. In-centrality denotes the association direction from the outer points to the central ones, which implies the agglomeration function of the central nodes in the network. Intermediary centrality reflects the intermediate transition role of a node’s associations with others, which implies an indirect connection among nodes in the whole network through the intermediate nodes. The calculation formulas are as follows:

3 $$C_{AD} = \frac{{\mathop \sum \nolimits_{i} \left( {C_{{AD_{max} }} - C_{{AD_{i} }} } \right)}}{{\left( {n - 1} \right)\left( {n - 2} \right)}},$$

4 $$C_{Out - D} = 2 \times \frac{{\mathop \sum \nolimits_{i} \left( {C_{{Out - D_{max} }} - C_{{Out - D_{i} }} } \right)}}{{\left( {n - 1} \right)\left( {n - 2} \right)}},$$

5 $$C_{In - D} = 2 \times \frac{{\mathop \sum \nolimits_{i} \left( {C_{{In - D_{max} }} - C_{{In - D_{i} }} } \right)}}{{\left( {n - 1} \right)\left( {n - 2} \right)}},$$

6 $${\text{C}}_{{{\text{AB}}}} = 2 \times \frac{{\mathop \sum \nolimits_{{\text{i}}} \left( {{\text{C}}_{{{\text{AB}}_{{{\text{max}}}} }} - {\text{C}}_{{{\text{AB}}_{{\text{i}}} }} } \right)}}{{\left( {{\text{n}} - 1} \right)^{2} \left( {{\text{n}} - 2} \right)}},$$

where, in formula (3), $C_{AD}$ represents the overall centrality, $C_{{AD_{max} }}$ represents the maximum value of the node degree in the network, and $C_{{AD_{i} }}$ indicates the centrality degree of node *i* in the whole network. Within the asymmetric network, the molecule in the formula doubles and the centrality values depend on the values of $P_{ij}$ or $P_{ji}$; $C_{Out - D}$ represents out-centrality, and $C_{In - D}$ is in-centrality. In formula (5), $C_{AB}$ represents the intermediary centrality, $C_{{AB_{max} }}$ represents the maximum value, and $C_{ABi}$ represents the intermediary degree of city *i* in the whole network. *N* is the number of cities in the network.

#### Calculate the parameter values of individual nodes in the network

The number of the current nodes directly connects to other nodes in the network. In an asymmetric network, in-node-centrality is expressed by the number of other nodes connecting to the current one, and it shows the direction of population flowing from other cities or towns into the current one. Out-node-centrality is represented by the number of other nodes to which the current node is connected, and it shows the direction of population flowing from the current city or town into other ones. Intermediary centrality indicates the role of the city through which the population flows, which plays an intermediate role in the network. It is generally believed that a city with a higher degree of centrality has more central power in the network and has stronger control and influence over the network. Accordingly, the formula for calculating the degree centrality of city *i* in the network is as follows:

7 $$C_{ADi} = \sum\limits_{j = 1,i \ne j}^{n} {G_{ij} },$$

8 $$C_{Out - i} = \sum\limits_{j = 1,i \ne j}^{n} {G_{ij} },$$

9 $$C_{{{\text{In - }}Di}} = \sum\limits_{j = 1,i \ne j}^{n} {G_{ji} },$$

10 $$C_{{A{\text{B}}i}} = \sum\limits_{a,b = 1,a \ne i,b \ne i}^{n} {G_{a - i - b} },$$

where $G_{ij}$ is the association between nodes *i* and *j*. If $P_{ij} > P_{i}$, then $G_{ij} = 1$, indicating that there is an association between nodes *i* and *j*, otherwise it is 0. ${C}_{Out-i}$ represents out-node-centrality, and ${C}_{In-Di}$ represents in-node-centrality. ${C}_{ABi}$ denotes intermediary centrality, which represents the frequency of association among other linked nodes through the current one. Under the graph theory, if a node in the network is on multiple shortest paths among other nodes, then this node is the core member and has greater mediating effect within the whole network.

#### Identification of internal subgroup

There is a small world consist of closely associated nodes in the network which is defined by the agglomeration group technology of the network analysis method, namely the iterative correlation convergence (CONCOR) and core-periphery structure analysis methods. Cities and towns are grouped internally via similar structures. The specific method for determining these groups is to iteratively calculate the correlation coefficient of each node and then group nodes with similar correlation coefficients together such that all nodes are divided into different groups.

#### The quadratic assignment procedure (QAP) method for finding the influencing factors on the urban network structure

In terms of the influencing factors impacting the network structure of the urban population, the traditional multiple linear regression is invalid for resolving the endogenous problem. This is because of the relations among the nodes, which cause strong endogenous among the nodes in addition to the endogenous variables. The quadratic assignment procedure (QAP) method in social network analysis can be used to explore the structural similarity among multiple networks, and it can find correlations among the dependent and independent variables to indicate the influencing factors of the network structure. The detailed approach is to convert the arrays of the dependent and independent variables into a long vector matrix and then compare the similarity among the corresponding lattice values of the network nodes. The significance of the coefficient is tested based on multiple row-column random permutations of the matrix data. This avoids the problems of multi-collinearity and endogeneity in traditional econometric regression analysis.

#### Dependent and independent network variables

The QAP method requires the selection of the dependent variable and corresponding explanatory factors to construct the independent network variables. We select the population network above as the dependent variable because the node size is determined by population size in the urban scale system. The independent variables are natural, transportation, and economic and social conditions. The reasons for selecting these are as follows.

#### Natural conditions

This involves two factors, altitude and distance, are denoted by the altitude of each node and the geographic proximity of the nodes adjacent to other counties. Altitude is a vital factor in the development of the Qinghai-Tibet Plateau, leading to the alpine and complex topography of the plateau and is directly related to factors including population distribution, which makes the ‘negative altitude effect^35^ significant. Factually, 99% of the population is distributed in the areas below 4500 m above sea level. Once the altitude exceeds 3800 m, the population density markedly decreases as the elevation rises. Meanwhile, there is an inverse exponential relationship between population distribution and topographic height^36^. As a result, we choose the altitude of each node as an influencing factor, and we use the altitude difference between two nodes to form a matrix to construct an independent network variable. Additionally, in terms of distance or geographical proximity, because of the dispersed distribution, the distance between towns is too much for the towns to be adjacent. However, the adjacent counties are sensitive to geographic proximity. The construction of the geographical proximity network is as follows: If the counties of two towns are adjacent, we set the value to 1, and 0 otherwise.

#### Transportation conditions

This variable is represented by the shortest driving time between two nodes. The sparse distribution of cities and towns in the Qinghai-Tibet Plateau has always been the main limiting condition for the development of the plateau. Building roads is difficult, and the transportation in the region is inefficient, which has cause the plateau to develop slowly and remain relatively static for a long time^37^. The evolution of the traffic network of the Qinghai-Tibet Plateau from 1976 to 2016 shows that the flow of the traffic network has greatly improved accessibility between the core towns and their surrounding areas^38^. Accordingly, we find that the role of the transportation network in the formation of the urban system of the Qinghai-Tibet Plateau is irreplaceable. Since the transportation in the Qinghai-Tibet Plateau is still dominated by highways, we capture the highway traffic patterns using the Gaode-Map software through python programming, and we calculate the shortest driving time between two cities and towns to build the traffic accessibility network.

#### Economic conditions

This variable is denoted with enterprise number by the altitude difference between two nodes. As an area lagging in terms of development, the main driving force of the Qinghai-Tibet Plateau’s urbanization comes from state financial subsidies, investment to form new towns, the relocation of coastal and inland factories and enterprises, and the establishment and expansion of administrative agencies^39^. It is difficult to achieve growth through an expansion of the market scale. However, the migration of enterprises to this area can create more employment opportunities, becoming the main factor for attracting people to towns. In an urban system, enterprises and people are more likely to migrate to economically efficient towns owing to the efficiency gradient among towns, thus changing the urban system structure^40^. This shows that the gradient of factors between two towns, represented by stock data, is prominent. To demonstrate the gradient change in the number of enterprises between two towns and reflect the close relationship between altitude and economic development in the areas, we divide the difference in the enterprise number by the altitude difference between two towns. We then obtain the matrix and construct an independent network variable.

#### Social conditions

This variable is determined by the two nodes in the same county unit. As a lagging area, the Qinghai-Tibet Plateau’s administrative force is powerful in terms of assigning resources. Administrative power is the main driving force behind urbanization in Tibet^41^. The different policy rules, development orientations, and competing interests on both sides of an administrative boundary impede the flow of people, exchange of goods, and sharing of resources. This intensifies market segmentation and hinders regional market integration^42,43^. Therefore, we choose the administrative boundary variant to construct the administrative correlation matrix: If towns are in the same county unit, then their relationship value is defined as 1, and 0 otherwise.

## Supplementary Information

Supplementary Table S1.

## Data Availability

The geographic dataset analyzed during the study was prepared by the author based on a map from the National Science and Technology Infrastructure: ‘National Earth System Science Data Center’[http://www.geodata.cn], are not publicly available due the institution requires formal non-profitable application for the usage of the data, but are available from the corresponding author on reasonable request.
