# Supplementary material for: Sustainable urban system structure evaluation in sparsely populated areas: case study of the Qinghai-Tibet Plateau in China
Source: Sci Rep. 2022 Sep 27;12:16067. doi: 10.1038/s41598-022-20367-5 (PMC9515182; doi:10.1038/s41598-022-20367-5)
Supplement: Supplementary file 1 — Supplementary Table S1. [file 41598_2022_20367_MOESM1_ESM.docx]

**Supplementary Table S1: Research units in the Qinghai-Tibet Plateau**

| **Huazangsi town, Tianzhu Tibetan autonomous county (2-1)** | Tianjiazhai town, Huangzhong county | Bazhou town, Minhe Hui and Tu autonomous county | **Jiezi town, Xunhua Salar autonomous county (1-7)** | Shuimo town, Wenchuan county | **Lhasa (3-8)** |
| --- | --- | --- | --- | --- | --- |
| Hongwansi town, Sunan Yugur autonomous county | **Ganhetan town, Huangzhong county (1-1)** | Manping town, Minhe Hui and Tu autonomous county | **Xihai town, Haiyan county (2-5)** | Fengyi town, Mao county | **Xigaze (3-9)** |
| **Jiangzi town, Jiangzi county (2-2)** | Gonghe town, Huangzhong county | Lierbao town, Minhe Hui and Tu autonomous county | **Maitang town, Jianzha county (2-6)** | Jinan town, Songpan county | **Shannan city (3-6)** |
| Qiaotou town, Datong Hui and Tu autonomous county | **Duo town, Huangzhong county (2-4)** | Xiamen town, Minhe Hui and Tu autonomous county | **Kangyang town, Jianzha county (1-2)** | Yongle town, Jiuzhaigou county | **Xining (3-10)** |
| Chengguan town, Datong Hui and Tu autonomous county | Guanlongkou town, Huangzhong county | **Weiyuan town, Huzhu Tu autonomous county (3-5)** | Heyin town, Guide county | Meixing town, Xiaojin county | **Haidong city (3-11)** |
| **Ta'er town, Datong Hui and Tu autonomous county (1-5)** | Lijiashan town, Huangzhong county | Bayan town, Hualong Hui autonomous county | **Mangqu town, Guinan county (1-8)** | Rangke town, Rangtang county | **Gaocheng town, Litang county (2-9)** |
| Dongxia town, Datong Hui and Tu autonomous county | **Chengguan town, Huangyuan county (1-6)** | Qunke town, Hualong Hui autonomous county | Cnwusu town, Dulan county | **Aba town, Aba county (2-7)** | **Niao town, Shiqu county (1-10)** |
| Huangjiazhai town, Datong Hui and Tu autonomous county | Pingan town, Pingan district | Yashiga town, Hualong Hui autonomous county | Laskui town, Hotan | Luqiao town, Luding county | **Luoxu town, Shiqu county (1-3)** |
| Changning town, Datong Hui and Tu autonomous county | Xiaoxia town, Pingan district | Gandu town, Hualong Hui autonomous county | **Yulong Kashi town, Hotan city (3-4)** | Lengqi town, Luding county | **Mengyi town, Shiqu county (1-4)** |
| Jingyang town, Datong Hui and Tu autonomous county. | Chuankou town, Minhe Hui and Tu autonomous county | Zaba town, Hualong Hui autonomous county | Bagqi town, Hetian county | Zhanggu town, Danba county | **Labrang town, Xiahe county (2-10)** |
| Duolin town, Datong Hui and Tu autonomous county | Gushan town, Minhe Hui and Tu autonomous county | Ansiduo town, Hualong Hui autonomous county | **Hanaizike town, Hotan county (1-9)** | Xianshui town, Daofu county | Chengguan town, Zhouqu county |
| **Xinzhuang town, Datong Hui and Tu autonomous county (2-3)** | Maying town, Minhe Hui and Tu autonomous county | **Jishi town, Xunhua Salar autonomous county (3-3)** | Baohe town, Weixi Lisu autonomous county | Xindu town, Luhuo county | Chengguan town, Lintan county |
| **Lushaer town, Huangzhong county (3-2)** | Guanting town, Minhe Hui and Tu autonomous county | Baizhuang town, Xunhua Salar autonomous county | Weizhou town, Wenchuan county | **Ganzi town, Ganzi county (2-8)** | **Liulin town, Zhuoni county (3-1)** |

Note: The bold text indicates towns selected by the hierarchical clock. 1-1 indicates that the hierarchical clock is numbered 1 in the first picture in Figure 3, and so on.
